# Comparative Analysis of Iron Homeostasis in Sub-Saharan African Children with Sickle Cell Disease and Their Unaffected Siblings

**DOI:** 10.3389/fped.2016.00008

**Published:** 2016-02-23

**Authors:** Selma Gomez, Aïssatou Diawara, Elias Gbeha, Philip Awadalla, Ambaliou Sanni, Youssef Idaghdour, M. Cherif Rahimy

**Affiliations:** ^1^Laboratoire de Biochimie et de Biologie Moléculaire, Faculté des Sciences et Techniques, University of Abomey-Calavi, Cotonou, Benin; ^2^Centre de Prise en charge Médicale Intégrée du Nourrisson et de la Femme Enceinte atteints de Drépanocytose, Faculté des Sciences de la Santé, University of Abomey-Calavi, Cotonou, Benin; ^3^Biology Program, Division of Science and Mathematics, New York University Abu Dhabi, Abu Dhabi, United Arab Emirates; ^4^Sainte-Justine Research Centre, Centre Hospitalier et Universitaire Sainte Justine, Montréal, QC, Canada

**Keywords:** iron homeostasis, sickle cell disease, anemia, iron deficiency, hepcidin, serum iron proteins, red blood cell indices

## Abstract

Iron is an essential trace element subject to tight regulation to ensure adequate running of biological processes. In sub-Saharan Africa where hemoglobinopathies are common, iron homeostasis is likely to be impaired by these conditions. Here, we assessed and compared key serum proteins associated with iron metabolism between sub-Saharan African children with sickle cell disease (SCD) and their unaffected siblings. Complete blood counts and serum concentrations of four key proteins involved in iron regulation (ferritin, transferrin, sTfR, and hepcidin) were measured for 73 children with SCD and 68 healthy siblings in Benin, West Africa. We found significant differences in concentration of transferrin, sTfR, and ferritin between the two groups. Hepcidin concentrations were found at unusually high concentrations but did not differ among the two groups. We found a significant negative correlation between hepcidin levels and both MCH and MCV in the SCD group and report that sTfR concentrations show a correlation with MCV and MHC in opposite directions in the two groups. These results highlight the unusually high levels of hepcidin in the Beninese population and the patterns of differential iron homeostasis taking place under SCD status. These results lay the foundation for a systematic evaluation of the underlying mechanisms deregulating iron homeostasis in populations with SCD or high prevalence of iron deficiency.

## Introduction

Sickle cell disease (SCD), an inherited disorder of hemoglobin (Hb) structure, is one of the most common severe disorders in the world (1). In 2010, sub-Saharan Africa accounted for two-third of SCD births worldwide, making it the most burdened region (2). In Benin, the under-five mortality rate of SCD was estimated at 15.5/1,000 in a cohort of patients benefiting from a tailored comprehensive clinical care program (CCCP) (3). SCD can also lead to severe complications in affected individuals, including significant homeostasis imbalance. Furthermore, imbalance in iron metabolism could be more accentuated in area where iron deficiency is a large health problem, such as in Benin (4, 5). Thus, it is of interest to study the patterns of iron homeostasis in a context in which both iron deficiency and SCD are common.

Regulation of iron supply is essential to ensure adequate running of biological processes, such as erythroid function, binding and transport of oxygen as well as cellular respiration, and DNA synthesis and reparation (6, 7). Iron deficiency in early childhood is associated with numerous adverse health effects, including immune, neurological, and cognitive development impairments that may be irreversible even after iron repletion (8, 9). Similarly, excess of iron is deleterious for health and has become a major cause of morbidity and premature mortality (10, 11). The total iron store in the human body is approximately 3–4 g and is mainly distributed in the Hb of mature red blood cells and developing erythroid cells. In normal conditions 1–2 mg of iron is absorbed daily and an equivalent amount is lost. There is no physiological system for iron elimination; however, iron metabolism, storage, and transport are tightly regulated by a key set of proteins (12, 13). The regulation of iron homeostasis depends on a complex feedback mechanism between body iron requirements and intestinal absorption. Thus, iron stores are subject to tight control from intake in the intestine to storage, turnover, redistribution, and mobilization in the body (13, 14).

In the last decades, there have been tremendous advances in deciphering the mechanisms of iron homeostasis in the body and the understanding of the interplay between serum proteins implicated in this process. Of these proteins, hepcidin, a small 25-amino-acid peptide, produced mainly by hepatocytes and secreted into the blood has been recognized as a key regulator in homeostasis. (15–17). Other key iron metabolism proteins in the serum include ferritin, transferrin, and soluble transferrin protein (sTfR). Polymorphisms in these proteins could affect iron metabolism (18, 19). However, such genetic variants have not been documented in Benin.

In this study, we compared key parameters of iron homeostasis between children with SCD (cases) and their unaffected siblings (control group) in the Republic of Benin, West Africa. We assessed and compared relevant hematological indicators and the serum proteins directly linked to iron metabolism between the two groups and evaluated the relationship between these hematological indicators and the serum iron proteins.

## Materials and Methods

### Subjects

A total of 141 children were enrolled in our study in 2010, including 73 children with SCD. These children were part of a large cohort of early diagnosis for SCD and CCCP (20) at the “Centre de Prise en charge Médicale Intégrée du Nourrisson et de la Femme Enceinte atteints de Drépanocytose” (CPMI-NFED) (National Institute dedicated to caring of Infants and Pregnant Women affected by SCD) in Cotonou, The Republic of Benin. The CCCP includes an intensive socio-medical intervention program that aims to diagnose SCD early and to attenuate the effects of the disease on children as they grow up. As part of the clinical follow-up program children orally received powdered generic containing 5 mg/kg/day of elemental iron with orange or lemon juice 30 min before meals. This iron supplementation was provided every 9 months for a period of 2 months. All children with SCD were sampled at least 1 month after they finished their iron supplementation regime and none of the unaffected children were taking iron during the course of the study. Of the 73 children with SCD, 15 were diagnosed with acute anemia and were transfused during the 4 months preceding sampling of which 8 were transfused once, two twice, one three time, and one six time. The control group consisted of 68 age-matched siblings of the recruited children with SCD. Approval for the study was obtained from the Ethical Committee of the Faculty of Health Sciences.

### Procedures

Children with SCD were enrolled in the study after informed consent was obtained. All children were sampled under steady state during regular follow-up visits in the morning between 8:00 and 11:00 a.m. Based on clinical assessment, age-matched unaffected siblings, without any history of acute illness at least 3 months prior to sampling, were brought by parents for sampling after informed consent was obtained. Complete blood counts (CBC) were immediately performed using an automatic blood cell analyzer (KX-21, Sysmex Corporation, Japan) (Table S1 in Supplementary Material). Sera were frozen and stored at −30°C for subsequent measurement of ferritin, transferrin, sTfR, and hepcidin.

Serum ferritin, transferrin, sTfR, and hepcidin concentrations were measured using their respective ELISA kits following the manufacturers’ recommended protocols: IBL International GMBH, Hamburg, Germany, for ferritin and Hepcidin and Prohormone, Modrice, Czech Republic, for transferrin and sTfR (Table S1 in Supplementary Material). The sTfR index was calculated as the ratio of log (sTfR) (nanogram/milliliter) to the log (serum ferritin) (nanogram/milliliter) as previously described (21). The reference ranges for iron homeostasis serum proteins indicating normal iron status were defined as follow: 25–283 ng/L for ferritin, 2–5 g/L for transferrin, 0.9–3.4 μg/mL for sTfR, 58.9–158.1 ng/mL for hepcidin, and 1.5 for the log (sTfR)/log (ferritin) ratio (22). For Hb, we used the pediatric cut off value defined by the World Health Organization (WHO) (23). The cut off values for the red blood cell indices were as follow: MCH < 25 pg and MCV < 75 fL.

### Data Statistical Analysis

Quantitative data were analyzed using Kruskal–Wallis, Wilcoxon and median non-parametric tests. A linear regression model was used to test for differential association in SCD patients and the control group between key hematological parameters and the assayed iron regulation proteins. Sex and age were included in regression analyses as covariates. These analyses test the hypothesis that proteins directly involved in iron homeostasis are *(a)* differentially regulated between SCD patients and their unaffected siblings and *(b)* influence iron homeostasis through regulation of MCH and MCV, two of the indices most correlated with iron regulation in the body. Hypothesis *(a)* was tested by comparing the mean and variance of the concentration of the assayed proteins between SCD patients and the controls. Hypothesis *(b)* was tested by comparing the relationship between both MCH and MCV, as dependent variables, and the assayed proteins in the SCD and control groups.

## Results

### Subject Characteristics

A total of 141 children, 73 SCD patients with Hb genotypes SS (53) or SC (20) and 68 unaffected siblings with Hb genotypes AA (21), AS (45), or AC (2) were recruited. The demographic, gender, and Hb characteristics of both groups are listed in Table 1. Age and sex distributions were not significantly different between the two groups (*p* = 0.1518 and *p* = 0.1595, respectively).

**Table 1:** **Characteristics of the study subjects**.

	SCD patients	Controls
**Patients, *n* (%)**	73 (100%)	68 (100%)
Male gender	43 (58.9%)	32 (47.1%)
Female gender	30 (41.1%)	36 (52.9%)
Median age, month (range)	36 (12–72)	33.5 (6–72)
**Hemoglobin (Hb) type, *n* (%)**		
HbSS	53 (72.6%)	0
HbSC	20 (27.4%)	0
HbAA	0	30 (30.9%)
HbAS	0	45 (66.2%)
HbAC	0	2 (2.9%)

### Hematological Profiles

Hematological details of patients in the steady state and controls are summarized in Table 2. As expected, the mean Hb concentration was significantly lower (*p* < 0.0001) in children with SCD (87.8 g/L) compared to the control group (110.0 g/L) with 87.7% of children having Hb values below the normal distribution. We note that in the control group 20.4% of children had a mean Hb concentration below the WHO’s threshold defining anemia in the corresponding age group (23). We also note that mean concentration for both red blood cell indices MCV and MCH were significantly reduced in the control group (*p* < 0.001) as 54 and 85% of children in the control group had MCV and MCH values below the cut off value of 75 fL and 25 pg, compared to 30 and 52% in the SCD group, respectively.

**Table 2:** **Hematological values in steady-state SCD patients and the controls**.

Indicators	SCD patients, mean ± SD	Controls, mean ± SD	*p*-value
Hemoglobin concentration (g/L)	87.8 ± 15.8	110.0 ± 9.0	<0.0001
MCV (fL)	79.2 ± 8.2	73.6 ± 6.1	<0.0001
MCH (pg)	25.0 ± 2.8	23.2 ± 2.4	<0.0001

### Serum Iron Protein Profiles in the Control and SCD Groups

**Figure 1:**
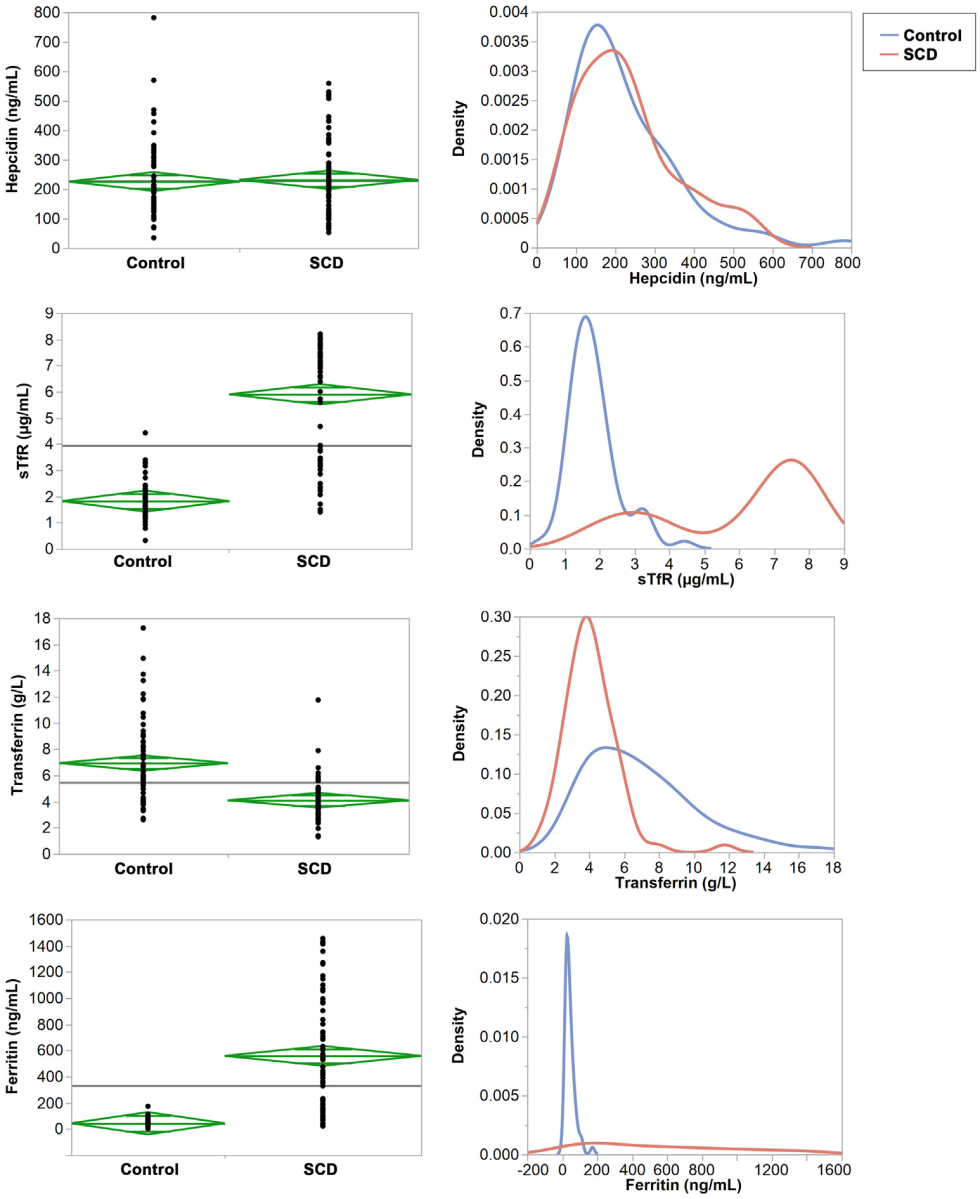
**Comparisons of means and variances of the four iron serum proteins in the control and SCD groups**. **Right panel**: the diamonds show the 95% confidence intervals and the horizontal black line shows the mean value across the entire set of individuals. **Left panel**: the densities show the distribution of each protein in the control and SCD groups.

Mean concentrations of transferrin, sTfR, ferritin, and the log (sTfR)/log (ferritin) index were statistically different (*p* < 0.001) between the SCD and control groups but hepcidin (*p* = 0.6487, Table 3). Testing for equal variances between SCD and control groups for the four proteins revealed that variance was significantly different except for hepcidin (Figure 1). In particular, we note the extreme case of ferritin that shows striking difference in variance between the two groups (Figure 1).

**Table 3:** **Iron homeostasis serum proteins profiles in SCD patients in the steady state and in controls**.

Proteins	SCD patients			Controls			*p*-value^c^
	Mean ± SD	Subjects with values above the cut-off^a^, *n* (%)	Median/interquartile range^b^	Mean ± SD	Median/interquartile range^b^	Subjects with values above the cut-off^a^, *n* (%)	
Transferrin (g/L)	4.1 ± 1.5	16 (21.9)	–	6.9 ± 3.1	–	46 (67.6)	<0.0001
sTfR (μg/mL)	5.9 ± 2.2	56 (76.7)	–	1.8 ± 0.7	–	5 (6.8)	<0.0001
Ferritin (ng/L)	558.9 ± 434.7	48 (65.8)	445.6/(174.8−933.3)	44.0 ± 28.9	37.6/(24.3−54.5)	0	<0.0001
sTfR index	1.5 ± 0.3	25 (34.2)	–	2.1 ± 0.9	–	58 (79.4)	<0.0001
Hepcidin (ng/mL)	231.6 ± 128.2	47 (64.4)	215.2/(127.3−287.4)	225.6 ± 135.1	192.9/(130.3−296.9)	41 (56.2)	0.6487

*^a^Cut-off values defined in Section “Materials and Methods*.”

*^b^Median and interquartile range for non-normally distributed proteins ferritin and hepcidin*.

*^c^Wilcoxon test, means comparisons*.

The mean values of transferrin and the log (sTfR)/log (ferritin) index were within the normal range (2–5 g/L and ≥1.5, respectively) in children with SCD (4.1 g/L and 5.9, respectively) whereas both were above the upper limit of normal values in the control group (6.9 g/L and 1.8, respectively). In addition, 67 and 79% of individuals in the control group have transferrin and sTfR index concentrations above the cut off. By contrast, in children with SCD, ferritin and sTfR levels were almost twofold above the upper limit of normal ferritin and sTfR (558.9 ng/L for ferritin and 5.9 μg/mL for sTfR), while in the control group both concentrations were within the normal ranges (59.0 ng/L for ferritin and 2 μg/mL for sTfR). We also compared ferritin concentration levels between SCD children with past history of blood transfusions and those who have never been transfused. The mean ferritin concentrations were not significantly different (*p* = 0.1072) between transfused and non-transfused children, suggesting that blood transfusion does not explain the observed high level of ferritin in children with SCD (Figure 1 in Supplementary Material). Finally, intriguingly in both SCD and control groups, mean hepcidin concentration were unexpectedly high (231.6 and 225.6 ng/mL, respectively) and above the upper limit of normal hepcidin values of 58.9–158.1 ng/mL. We should also note that within the control group, mean transferrin concentrations were found to be significantly different between AA and AS/C genotypes (*p* = 0.006) (Figure 2 in Supplementary Material).

### Correlation between the Four Iron Homeostasis Serum Proteins and MCV and MCH

**Figure 2:**
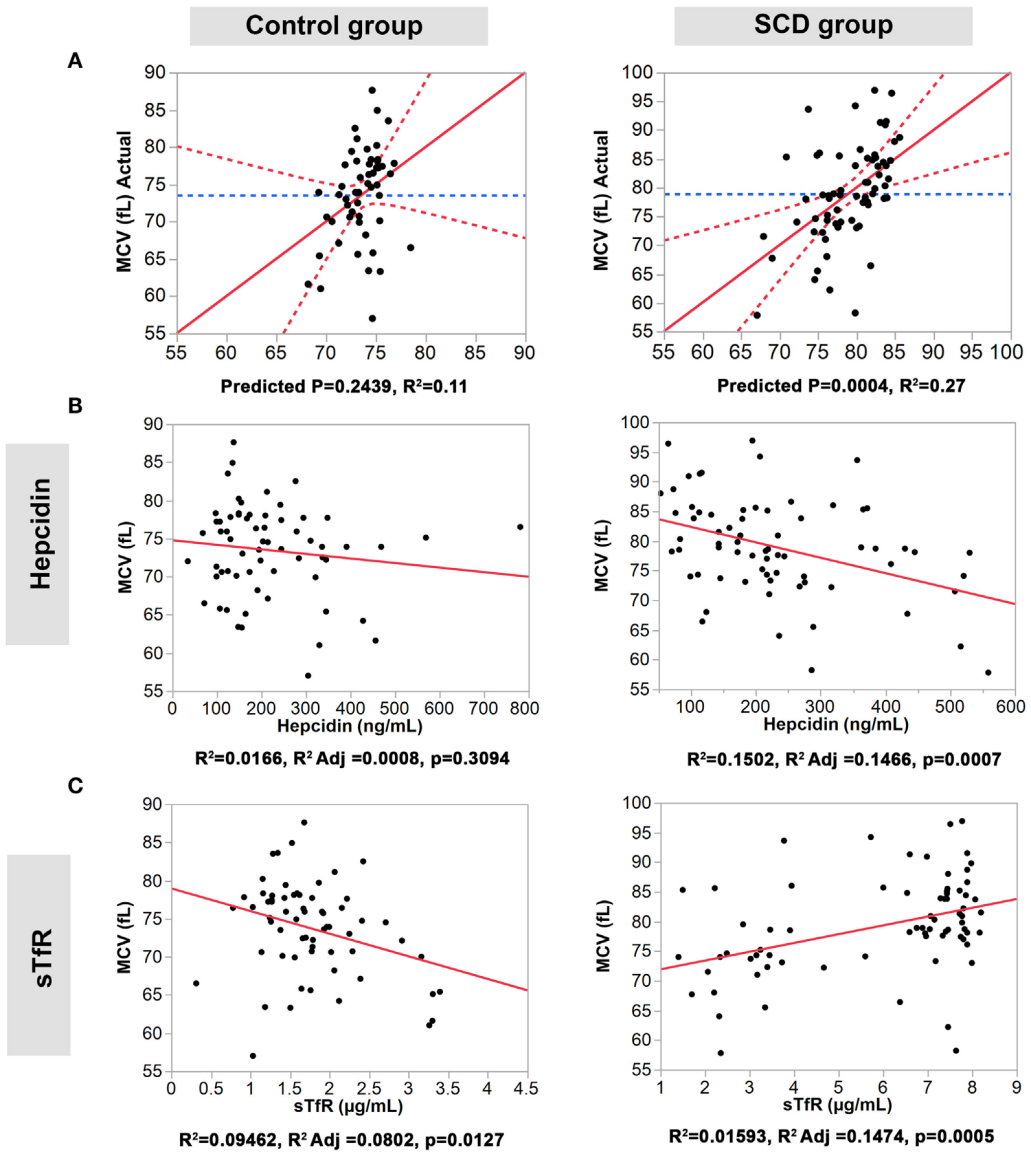
**Association of hepcidin and sTfR with MCV in the control and SCD groups**. **(A)** Full multivariate models (control group, left panel, and SCD group, right panel). **(B,C)** Univariate models testing the association of each protein (control group, left panels, and SCD group, right panel). The red solid line shows the line of fit, the red dashed line represents the 95% confidence curves and the blue dashed line shows the horizontal mean reference that represents the null hypothesis.

**Figure 3:**
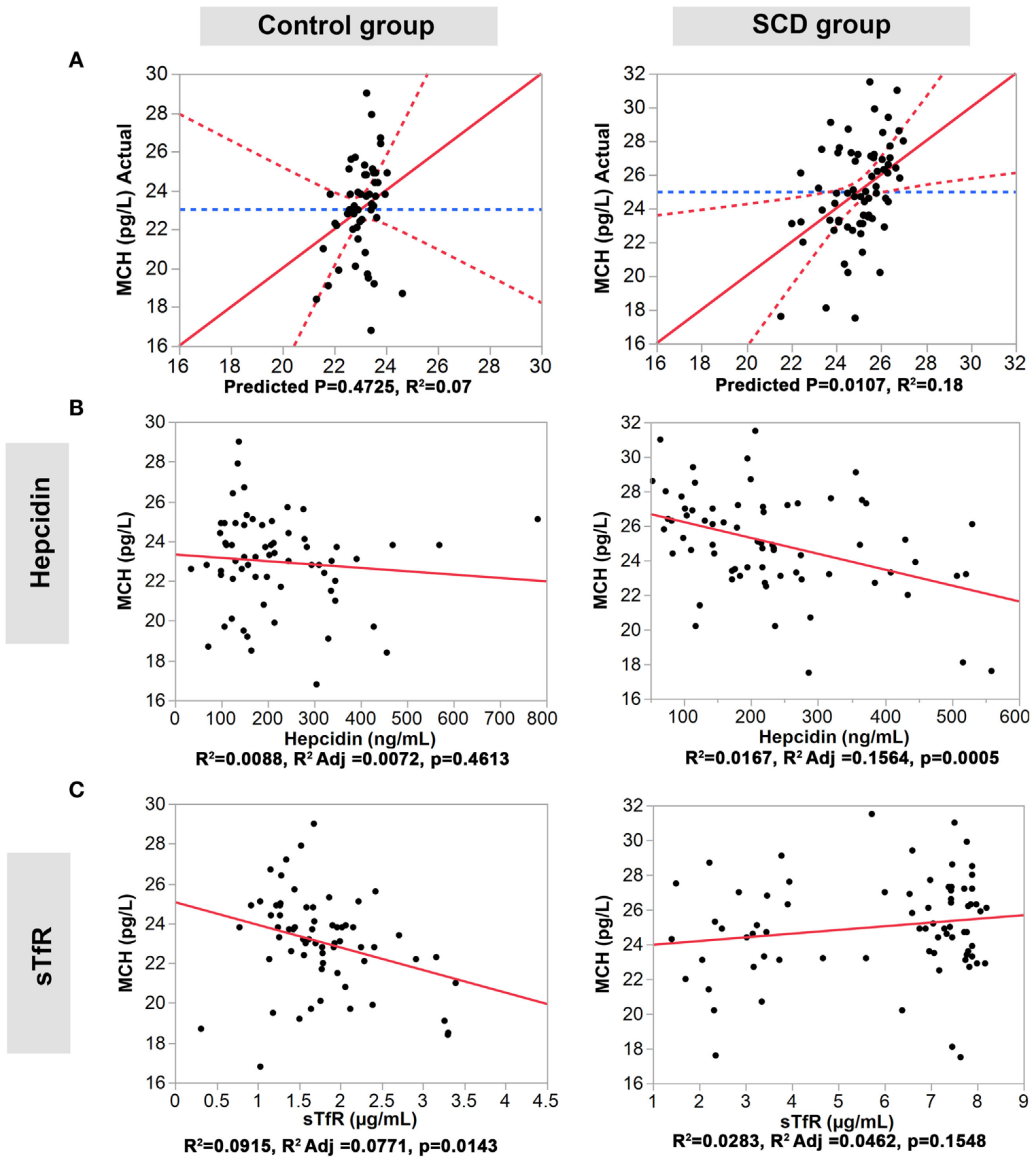
**Association of hepcidin and sTfR with MCH in the control and SCD groups**. **(A)** Full multivariate models (control group, left panel, and SCD group, right panel). **(B,C)** Univariate models testing the association of each protein (control group, left panels, and SCD group, right panel). The red solid line shows the line of fit, the red dashed line represents the 95% confidence curves and the blue dashed line shows the horizontal mean reference that represents the null hypothesis.

We performed multiple regression analyses to examine the relationship between the four serum iron proteins and MVC and MCH levels in SCD and control groups. First, we constructed a multivariate statistical model that includes all four serum proteins assayed against MCV or MCH. The predicted against actual Y plots (Figures 2 and 3) show that the models fit best in the SCD group (*p* = 0.0004 and *p* = 0.0107, respectively) relative to the control group where the fit is not statistically significant (*p* = 0.2439 and *p* = 0.4725, respectively). Univariate analyses showed that hepcidin concentrations were negatively correlated with MCV and MCH levels in the SCD group (Pearson correlation *r*^2^ = 0.1502, *p* = 0.0007 and *r*^2^ = 0.0167, *p* = 0.0005, respectively, Figures 2 and 3). Also, sTfR concentrations show opposite trends in the two groups being negatively correlated with MCV and MCH in the control group (*r*^2^ = 0.0946, *p* = 0.0127 and *r*^2^ = 0.0915, *p* = 0.0143, respectively) and positively correlated with MCV and MCH in the SCD group (*r*^2^ = 0.1593, *p* = 0.0005 and *r*^2^ = 0.0283, *p* = 0.1548, respectively) (Figures 2 and 3). We note that transferrin and ferritin show the same trend as sTfR in both groups but it is not statistically significant (Figure 3 in Supplementary Material). These findings show that the association between key hematological indicators (i.e., MCH and MCV) and hepcidin and sTfR is altered under the SCD condition.

To test for the effect of blood transfusion of the observed differences, we removed from the analysis the study participants who received blood transfusion and observed no significant differences in the results. To test for differences between genotypic classes within the control group, we performed the association tests by genotype (AA and AS/C groups). Of the four proteins investigated and although not statistically significant, hepcidin and ferritin concentrations showed two opposite trends in the two groups, being positively correlated with MCV and MCH in the AA group and negatively correlated in the AS/C group (Figure 4 in Supplementary Material). These findings demonstrate that the observed differences in hepcidin and ferritin between the SCD and control groups detailed above are more pronounced when SCD patients are compared to the AA genotype group.

## Discussion

Sickle cell disease is common throughout much of sub-Saharan Africa. In Benin, SCD is associated with high rate of childhood mortality and morbidity (3). Although many aspects of SCD are studied, reports on proteins involved in iron homeostasis in children with SCD are scarce. Given the above, we conducted this study to document the patterns of iron metabolism in young children with and without SCD by examining hematological indices and four key serum iron metabolism-associated proteins.

The hematological indices assessed closely in our study are Hb, MCV, and MCH. Subjects with SCD had significantly lower levels of Hb compared to the controls. This observation was expected considering the physiopathology of the disease. We also note that 20.4% control children had Hb levels below the normal range but this was not associated with the sickle cell trait phenotype. In contrast to other reports (24, 25), SCD subjects in our study had higher levels of RBC indices compared to the control group. This observation could be due to the effect of the follow-up clinical program, part of which children take iron supplements (13). These low values could be explained partly by iron deficiency, one of the main causes of anemia (26, 27). Rahimy et al. (5) previously showed that iron deficiency was a predominant condition in healthy young children in Benin. Our results are consistent with this finding, suggesting that the hematological levels in our control group are in line with the hematological profiles reported in the general pediatric population in Benin.

To assess the effect of SCD status on indicators and parameters associated with iron homeostasis (22, 28, 29), we compared the concentration of four serum iron proteins (transferrin, ferritin, sTfR, and hepcidin) and the log (sTfR)/log (ferritin) index between SCD subjects and controls. Iron balance is regulated by modulating the rate of erythropoiesis and by the amount of iron stores (30). When iron is deficient, the concentration of transferrin and sTfR increases, while that of ferritin synthesis and hepcidin declines (31, 32). In contrast to the control group, the SCD group had elevated concentrations of ferritin and sTfR and lower levels of transferrin and log (sTfR)/log (ferritin) index. Such elevated concentrations of ferritin and sTfR are usually associated with iron overload and have been reported in the case of blood transfusion (33, 34). However, this scenario is unlikely in our study as we do not observe a significant association between blood transfusion history and ferritin concentration in our cohort, suggesting the presence of an excess of intravascular hemolysis causing an increase of iron absorption as previously reported (35). Elevated levels of ferritin contrasted with normal levels of transferrin and sTfR and a normal log (sTfR)/log (ferritin) index. This observation is intriguing and warrants further investigation to unravel the underlying mechanisms.

Intriguingly, we observed high levels of hepcidin in both SCD group and controls with approximately 65 and 56% of individuals in SCD and control group, respectively, showing hepcidin levels above the upper limit of the normal range. This observation suggests that the high level of hepcidin is a common trait in Beninese children. We also observed a significant negative correlation between hepcidin levels and both MCH and MCV only in the SCD group and an opposite trend for sTfR and both MCH and MCV in the control group (negative correlation) and in the SCD group (positive correlation). We also assessed the potential effect of sickle cell trait genotype on iron homeostasis proteins by stratifying the control group by genotype (AA versus AS/C) in our association analysis. Several studies have highlighted the link between SCD trait genotypes and conditions, such as systemic inflammation (36) and oxidative stress (37). Our results show differences between AA and AS/C genotypes and demonstrate that the opposite trends observed for hepcidin and ferritin between SCD patients and the controls are driven by the AA individuals. Normal to low levels of hepcidin in SCD have been previously reported (38, 39). However, our results do not support the generalization of these findings. This observation hints to the possibility that the effect of other variables modulating the production of hepcidin might be in play in our cohort or in West African populations in general as indicated by the unusually high levels of hepcidin even in the unaffected group. High levels of hepcidin under severe iron deficiency have only been reported in pathological conditions, such as rare hepatic adenomas and familial iron-refractory iron deficiency anemia (40, 41). In our study population, it is unlikely that the observed patterns results from pathological conditions, given our knowledge of medical history of the study subjects and given that the trend is observed in both disease and control groups. The observed relationship between hepcidin and MCH and MCV in populations such as Benin where iron deficiency is prevalent can also be modulated by diet and/or selective pressure to maintain low levels of iron in the body against infectious agents that require high levels of iron (42–45). We hypothesize that these factors contribute to the negative correlation we observe between hepcidin and both MCH and MCV, an effect exacerbated under SCD status. However, further investigations are required to confirm these hypotheses. Our study also highlights the unusually high levels of hepcidin in the Beninese population and the differential patterns of iron homeostasis through the association between hepcidin levels and key hematological parameters. Furthermore, SCD patients are more likely to suffer from nutritional deficiencies caused by increased metabolic requirements (46, 47). Polymorphisms in genes coding for iron regulation proteins can also modulate the mechanisms of iron metabolisms (48). Future studies should account for these variables that can alter the expression and/or function of the key iron metabolism proteins.

These results lay the foundation for a systematic evaluation of the underlying mechanisms deregulating iron homeostasis in populations with SCD or high prevalence of iron deficiency.

## Author Contributions

MCR and AS designed the study, MCR and YI supervised the study, EG and PA provided reagents, SG performed the experiments, AD analyzed the data, and MCR, YI, and AD wrote the manuscript.

## Conflict of Interest Statement

The authors declare that the research was conducted in the absence of any commercial or financial relationships that could be construed as a potential conflict of interest.
