# Supplementary material for: Comparative Analysis of Iron Homeostasis in Sub-Saharan African Children with Sickle Cell Disease and Their Unaffected Siblings
Source: Front Pediatr. 2016 Feb 23;4:8. doi: 10.3389/fped.2016.00008 (PMC4762986; doi:10.3389/fped.2016.00008)
Supplement: Supplementary file 2 [file Data_Sheet_1.docx]

**Supplementary Material**

**Comparative analysis of iron homeostasis in sub-Saharan African children with sickle cell disease and their unaffected siblings**

Selma Gomez^#^, Aïssatou Diawara^#^, Elias Gbeha, Philip Awadalla, Ambaliou Sanni, Youssef Idaghdour* and M Cherif Rahimy*

^#^Contributed equally to this work

*Correspondence:

M Cherif Rahimy

[mrahimy@bj.refer.org](mailto:mrahimy@bj.refer.org), [mrahimy2@yahoo.fr](mailto:mrahimy2@yahoo.fr)

Youssef Idaghdour

[youssef.idaghdour@nyu.edu](mailto:youssef.idaghdour@nyu.edu)

**Supplementary Table1. Characteristics of the study subjects.**

**Supplementary Figure 1. Comparisons of ferritin concentrations in transfused and non-transfused SCD children.** Diamonds show the 95% confidence intervals and the horizontal black line shows the mean value across the entire set of individuals.

**Supplementary Figure 2. Distributions of serum iron proteins in the two genotypic classes (AA and AS/C) of the control group**

Box plots show the 95% confidence intervals and the horizontal line shows the mean value across the entire set of individuals.* p-value= 0.006

**Supplementary Figure 3. Association of transferrin and ferritin with MCV and MCH in the control and SCD groups**

Univariate models testing the association between MCV and MCH, and transferrin and ferritin in the control and SCD groups. The red solid line shows the line of fit.

**Supplementary Figure 4. Association of hepcidin and ferritin with MCV and MCH in the two genotypic classes (AA and AS/C) of the control group**

Univariate models testing the association between hepcidin and ferritin, and MCV and MCH.
