# Supplementary figures and images for: Comparative Analysis of Iron Homeostasis in Sub-Saharan African Children with Sickle Cell Disease and Their Unaffected Siblings
Source: Front Pediatr. 2016 Feb 23;4:8. doi: 10.3389/fped.2016.00008 (PMC4762986; doi:10.3389/fped.2016.00008)

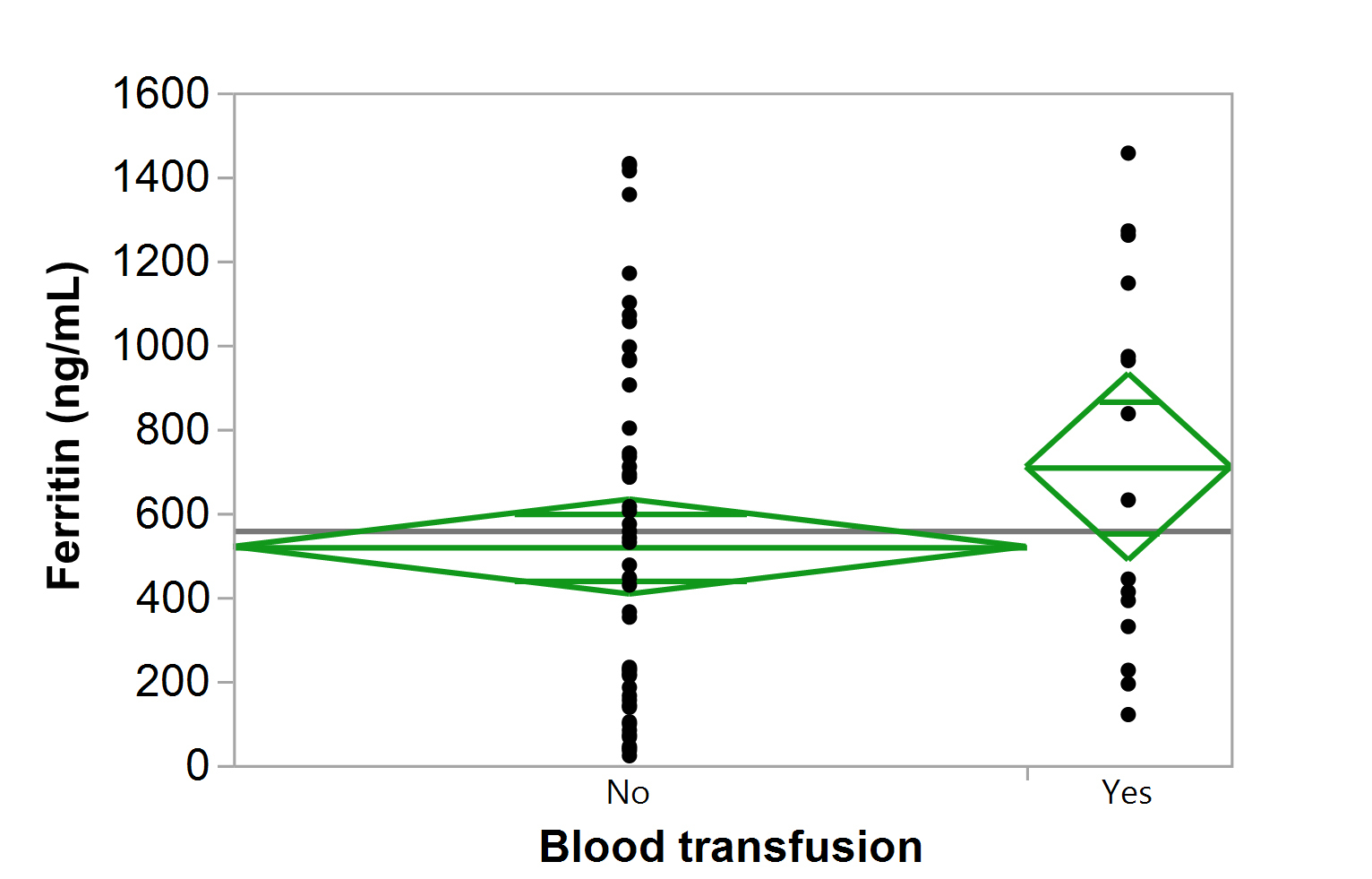

Supplement: Supplementary file 3 [file Image_1.jpg]

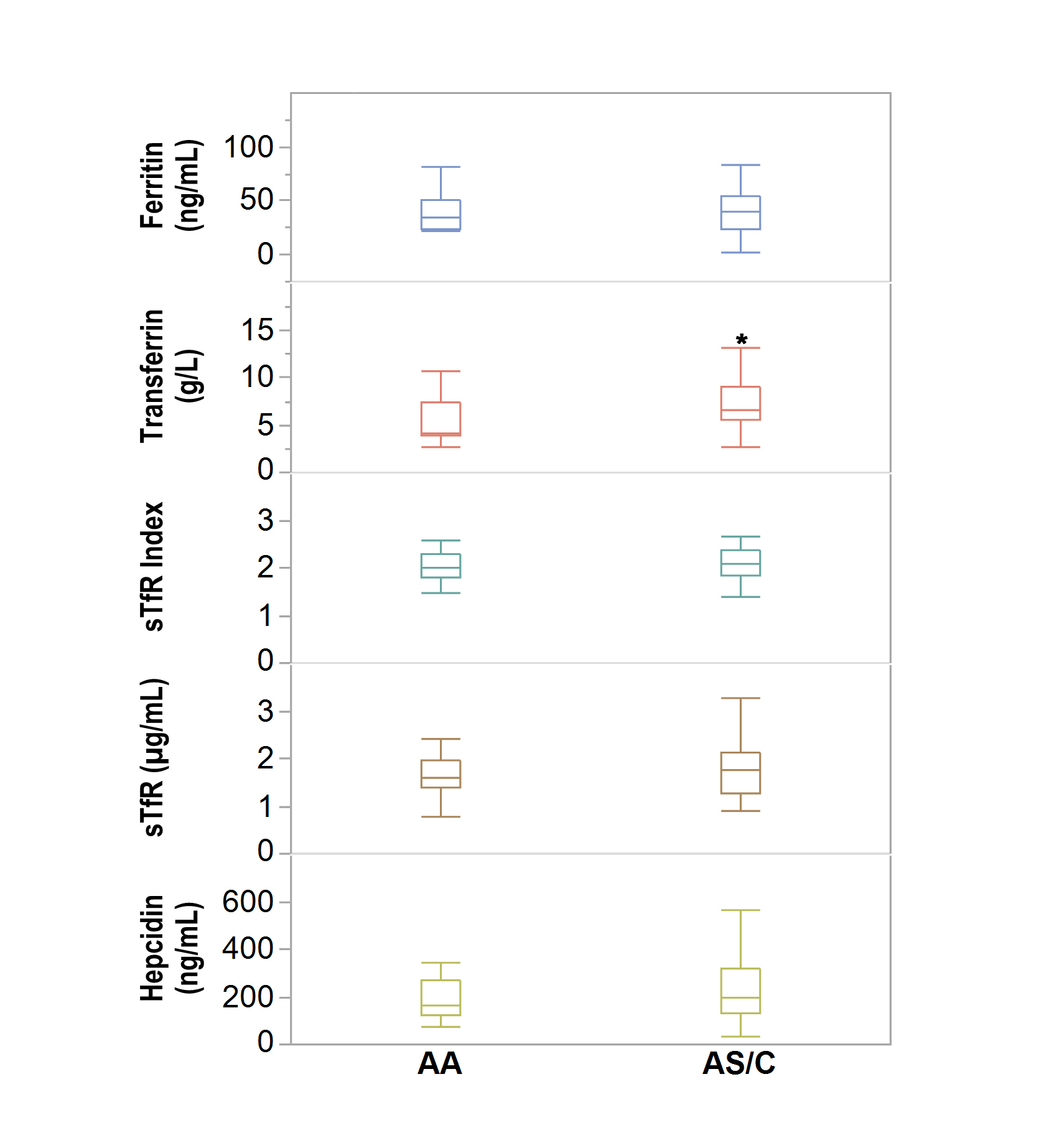

Supplement: Supplementary file 4 [file Image_2.jpg]

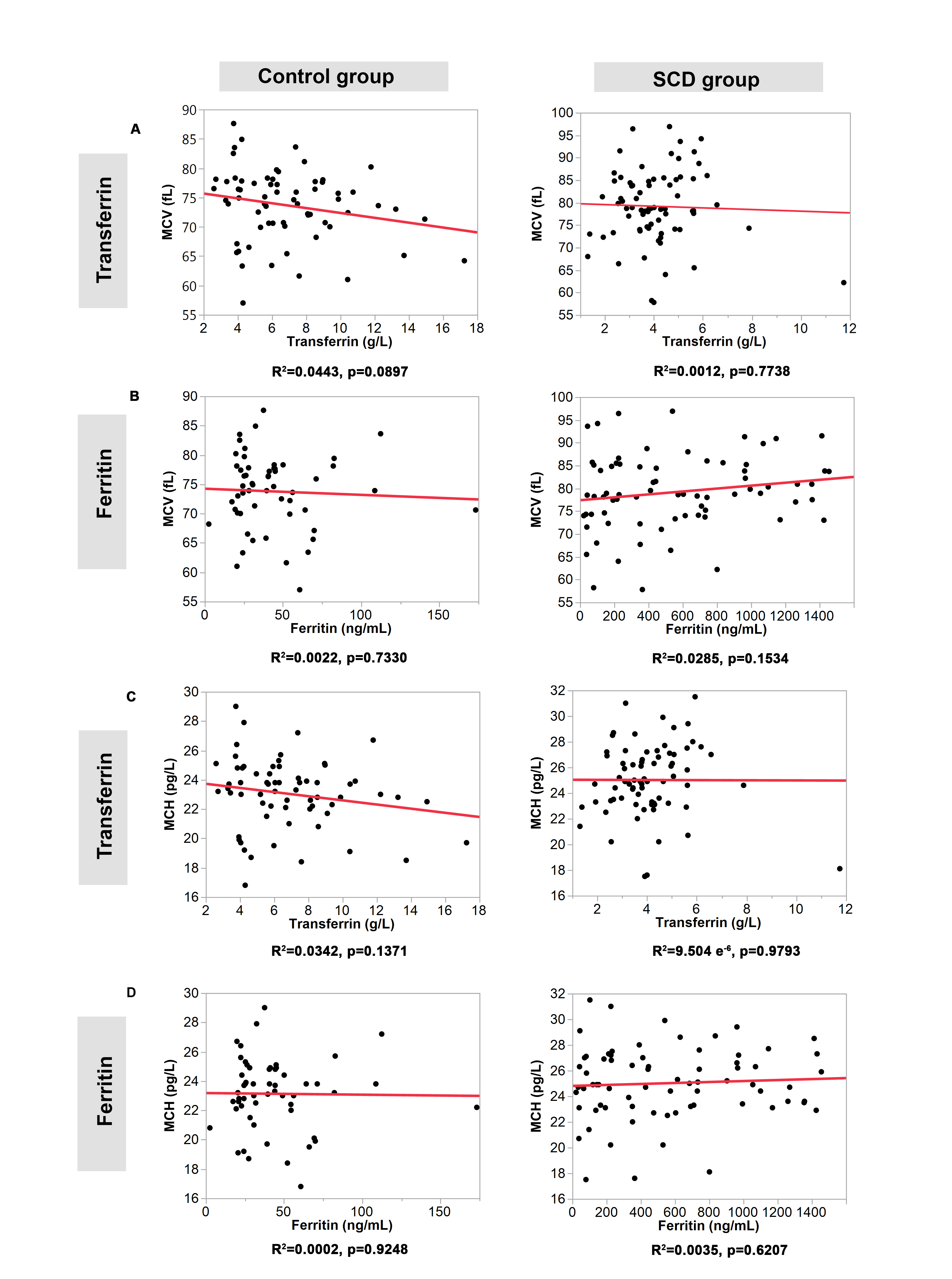

Supplement: Supplementary file 5 [file Image_3.jpg]

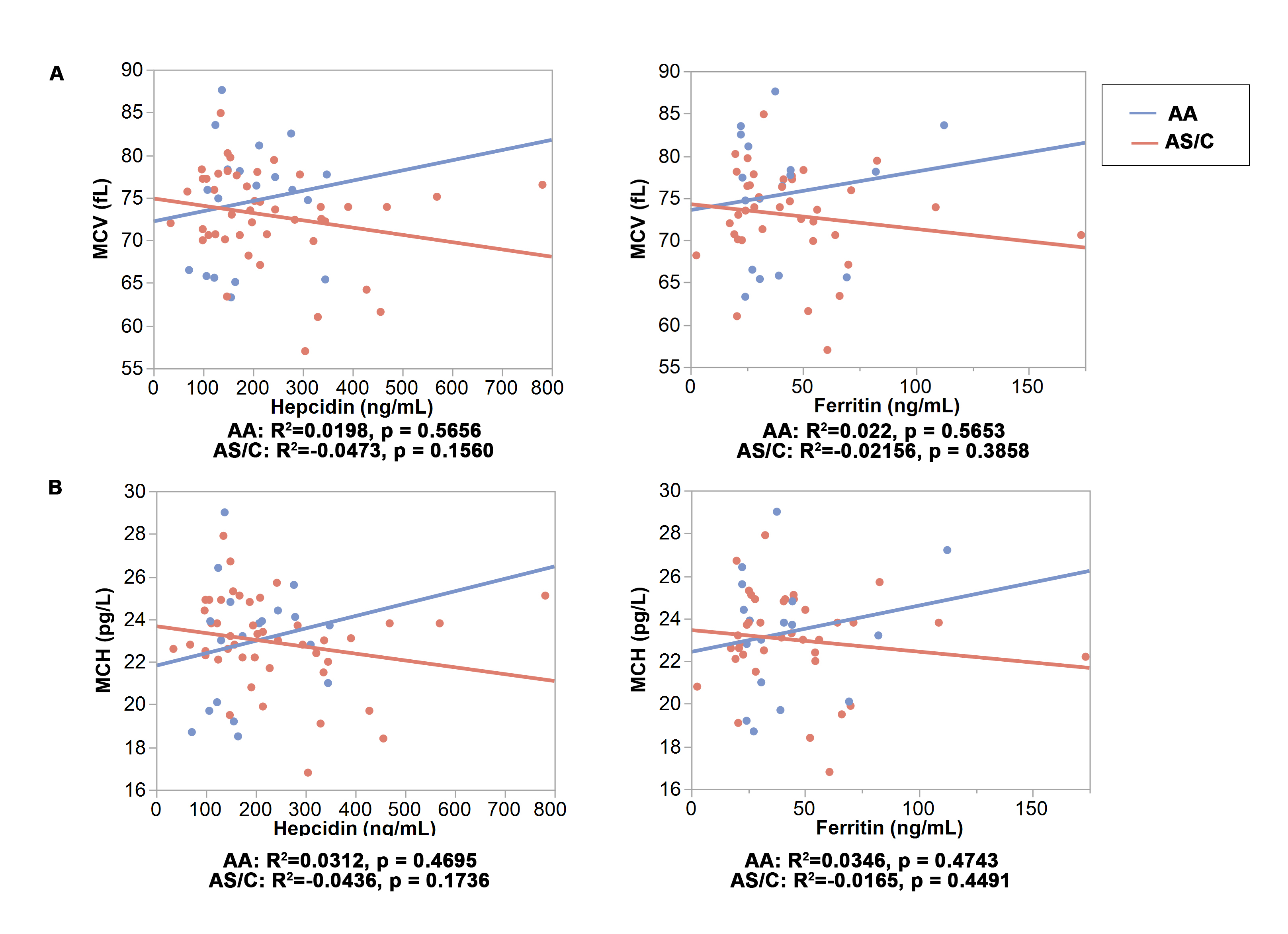

Supplement: Supplementary file 6 [file Image_4.jpg]
